# Structural Modifications of the Quinolin-4-yloxy Core to Obtain New *Staphylococcus aureus* NorA Inhibitors

**DOI:** 10.3390/ijms21197037

**Published:** 2020-09-24

**Authors:** Rolando Cannalire, Gianmarco Mangiaterra, Tommaso Felicetti, Andrea Astolfi, Nicholas Cedraro, Serena Massari, Giuseppe Manfroni, Oriana Tabarrini, Salvatore Vaiasicca, Maria Letizia Barreca, Violetta Cecchetti, Francesca Biavasco, Stefano Sabatini

**Affiliations:** 1 Department of Pharmaceutical Sciences, Università degli Studi di Perugia, via del Liceo 1, 06123 Perugia, Italy; rolando.cannalire@unina.it (R.C.); andrea.astolfi@unipg.it (A.A.); serena.massari@unipg.it (S.M.); giuseppe.manfroni@unipg.it (G.M.); oriana.tabarrini@unipg.it (O.T.); maria.barreca@unipg.it (M.L.B.); violetta.cecchetti@unipg.it (V.C.); stefano.sabatini@unipg.it (S.S.); 2 Department of Pharmacy, University of Napoli “Federico II”, via D. Montesano 49, 80131 Napoli, Italy; 3 Department of Life and Environmental Sciences, Università Politecnica delle Marche, via Brecce Bianche, 60131 Ancona, Italy; g.mangiaterra@staff.univpm.it (G.M.); n.cedraro@pm.univpm.it (N.C.); s.vaiasicca@pm.univpm.it (S.V.)

**Keywords:** antimicrobial resistance, NorA, efflux pump inhibitors, *Staphylococcus aureus*, scaffold hopping, quinoline, pharmacophore model, virtual screening, antimicrobial resistance breakers (ARBs)

## Abstract

Tackling antimicrobial resistance (AMR) represents a social responsibility aimed at renewing the antimicrobial armamentarium and identifying novel therapeutical approaches. Among the possible strategies, efflux pumps inhibition offers the advantage to contrast the resistance against all drugs which can be extruded. Efflux pump inhibitors (EPIs) are molecules devoid of any antimicrobial activity, but synergizing with pumps-substrate antibiotics. Herein, we performed an *in silico* scaffold hopping approach starting from quinolin-4-yloxy-based *Staphylococcus aureus* NorA EPIs by using previously built pharmacophore models for NorA inhibition activity. Four scaffolds were identified, synthesized, and modified with appropriate substituents to obtain new compounds, that were evaluated for their ability to inhibit NorA and synergize with the fluoroquinolone ciprofloxacin against resistant *S. aureus* strains. The two quinoline-4-carboxamide derivatives **3a** and **3b** showed the best results being synergic (4-fold MIC reduction) with ciprofloxacin at concentrations as low as 3.13 and 1.56 µg/mL, respectively, which were nontoxic for human THP-1 and A549 cells. The NorA inhibition was confirmed by SA-1199B ethidium bromide efflux and checkerboard assays against the isogenic pair SA-K2378 (*norA*++)/SA-K1902 (*norA*-). These *in vitro* results indicate the two compounds as valuable structures for designing novel *S. aureus* NorA inhibitors to be used in association with fluoroquinolones.

## 1. Introduction

Antimicrobial resistance (AMR) represents a complex global health challenge due to its natural insurgence and rapid spread caused by the use and misuse of antimicrobial agents in humans and animals [1,2,3,4]. The magnitude of the problem worldwide, the impact of AMR on human health and on costs for the health-care sector are still largely unknown [5]. Estimations report that yearly in Europe about 31100 people die as a result of multidrug-resistant (MDR) microbial infections, burdening on the European Union costs for €1.5 billion annually. Recent reports estimate that if no action is taken, by 2050 AMR will cause up to 10 million annual deaths globally [6,7]. To date, the selective pressure on microorganisms exerted from the boundless employment of antibiotics and the complexities of return on investment for pharmaceutical companies has meant that AMR is a common-place amongst microbial pathogens and for all antibiotic classes [8]. The urgent need to obtain new antibiotics does not leave room to failure, thereby directing investment and funds towards the chemical modification of known antibacterials typically acting on few antibacterial targets. However, a long-term strategy to overcome the early insurgence of AMR has necessarily to turn the attention on novel chemical scaffolds acting with new mechanisms of action. Moreover, a challenge strategy relies on the approach of hitting and halting the mechanisms responsible of the AMR. The fascinating idea to freeze resistance would allow to rescue failing antibiotics, thereby restoring our antimicrobial armamentarium. The molecules able to counteract AMR have been named in different ways: adjuvant molecules, helper compounds or antimicrobial resistance breakers (ARBs) [9,10,11,12]. All are characterized by the lack of antimicrobial activity and the ability to synergize with known antimicrobials, which leads to the recovery of their effectiveness against resistant strains. Since microorganisms mainly evolve resistance only for compounds exerting bactericidal or bacteriostatic effects, the lack of the antimicrobial activity of ARBs seems to be a strength for their potential use [13].

Bacterial efflux pumps are involved in essential microbial functions including the extrusion of noxious agents such as antibacterials, thus the development of efflux pump inhibitors (EPIs) represents a promising strategy to counteract AMR [14]. Based on the amino acid sequence similarities, predicted secondary protein structures and phylogenetic relationship, microbial efflux pumps are divided into six different families: (i) ATP-Binding Cassette (ABC) superfamily, (ii) Major Facilitator Superfamily (MFS), (iii) Multidrug And Toxic compound Extrusion (MATE) superfamily, (iv) Small Multidrug Resistance (SMR) superfamily, (v) Proteobacterial Antimicrobial Compound Efflux (PACE) family, and (vi) Resistance-Nodulation-cell Division (RND) superfamily [15]. According to the energy source used to extrude substrates, efflux pumps fall in two classes depending on whether they use ATP hydrolysis or the proton motive force (PMF) to drive drug transport [16].

The transmembrane protein NorA, belonging to MFS and using a PMF as energy source, is responsible for the extrusion of different chemicals (including the fluoroquinolone ciprofloxacin (CPX) and the dye ethidium bromide (EtBr)) and it is the most studied efflux pump in *Staphylococcus aureus*, a Gram-positive bacterium recently grouped among the ESKAPE pathogens [17,18,19,20]. Methicillin resistant *S. aureus* (MRSA) is one of the main nosocomial pathogens and represents an important challenge to the human health being both responsible of life-threatening infections, such as septic shock, endocarditis, osteoarticular infections and pneumonia and usually resistant to different classes of antibiotics [21].

**Figure 1:**
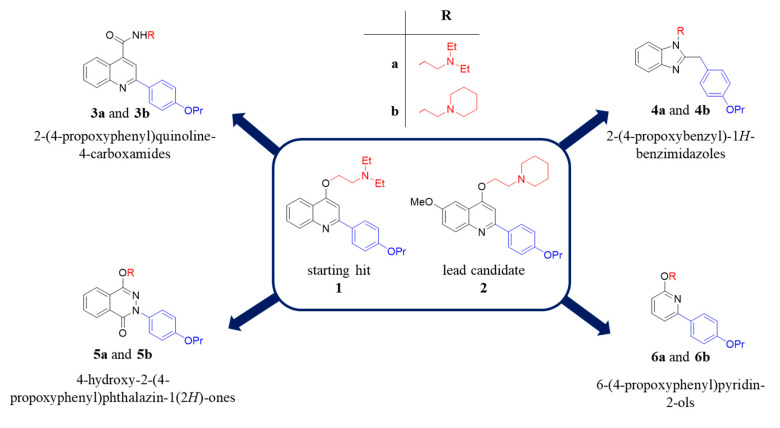
Chemical structures of the starting hit **1** and the lead candidate **2** and new synthesized compounds emerged from the *in silico* scaffold hopping approach.

Along the years, many NorA EPIs have been discovered by using three different approaches: (i) screening of natural or synthetic compound libraries, (ii) drug repurposing and (iii) designing and synthesizing new compounds based on phenotypic screenings [22]. On the other hand, the lack of a NorA three-dimensional structure and of biophysical/biochemical assays using the isolated protein has strongly hampered the structure-based drug design and the identification of potent NorA EPIs, which never reached clinical trials. However, some chemical classes of NorA EPIs [22], such as indoles [23,24,25], quinolines [26,27], boronic acids [28,29], chalcones [30], and piperine derivatives [31,32], exhibited promising results. From our side, we focused our efforts on the quinolin-4-yloxy scaffold and widely investigating the SAR, thus identifying some important features required for NorA inhibition, i.e., the propoxyphenyl group at C-2 position and the alkylamino chain linked to the oxygen at C-4. Consistently, the derivative **1** and an optimized 6-OMe analogue **2** (Figure 1), possessing these chemical requirements, are potent NorA EPIs [26,33]. In particular, derivative **2** represents the lead candidate of the quinolin-4-yloxy class being the most potent NorA EPI reported so far able to synergize with CPX at very low concentrations (0.78 µg/mL) against the resistant *S. aureus* SA-1199B strain (*norA*+/GrlA mutation) [34], without showing nonspecific effects during NorA inhibition (such as bacterial membrane depolarization) and resulting not active in *norA*-deleted *S. aureus* strains. In addition, it displayed promising pharmacokinetic properties and poor toxicity at high concentrations against different human cell lines [26].

Herein, to enrich the array of NorA inhibitors, we performed a scaffold hopping strategy of the quinolin-4-yloxy backbone, using as template the starting hit **1** (Figure 1) [33]. Combining scaffolds extracted by Food and Drug Administration (FDA) approved drugs, we built a scaffold library to replace quinolin-4-yloxy core and introduced the chemical substituents that in our quinolines gave the best NorA inhibition, i.e., the propoxyphenyl group at C-2 position and the alkylamino chain linked to the oxygen at C-4. The new virtual library was analysed by performing virtual screening experiments using our previously constructed pharmacophore models for NorA inhibitors to select the most interesting derivatives [35]. Only the scaffolds able to (i) correctly reproduce the mutual positions of the two above mentioned substituents and (ii) accept the two chemical functionalities in different positions were retained, thus generating about 6000 new virtual compounds. Four different scaffolds functionalized with the chemical key requirements for NorA inhibition have been selected based on the fitness values on the pharmacophore models and the chemical accessibility. In particular, the four selected scaffolds were used to synthesize eight new derivatives (**3a**, **3b**, **4a**, **4b**, **5a**, **5b**, **6a** and **6b**—Figure 1) that were biologically evaluated as NorA EPIs.

## 2. Results and Discussion

### In Silico Scaffold Hopping

We have recently developed two common-features pharmacophore models (hereafter called ModB and ModC) for NorA EPIs, which allowed the identification of FDA-approved drugs endowed with potent inhibitory activity [35]. Among the 3D chemical features, a positive charge appeared to be the key element in the discrimination between active and inactive compounds for both models. It should be noted that many quinoline derivatives were used to develop and validate the pharmacophore hypothesis, underlining an important role of the protonable moieties located in position 4 of the quinoline scaffold, with the ethyl-*N*,*N*-diethylamine (e.g., compound **1**) and 1-ethylpiperidine (e.g., compound **2**) groups being generally associated with the most interesting results in terms of EPI activity and physico-chemical properties.

**Figure 2:**
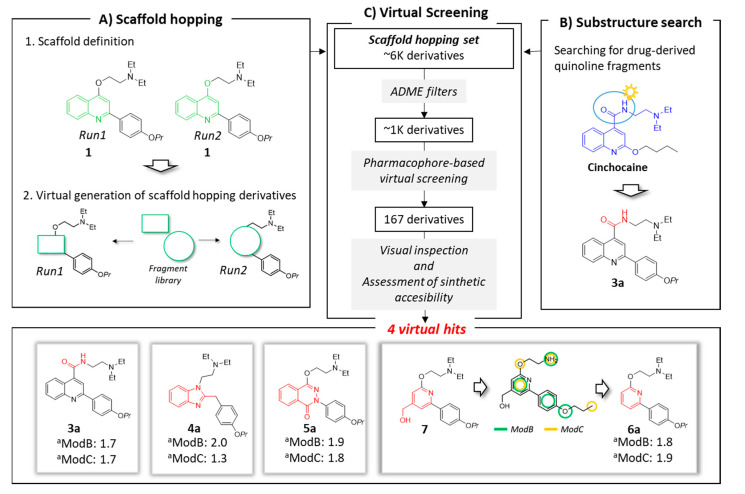
Schematic overview of applied *in silico* workflow. Panel A: two scaffold hopping runs were performed. In each run, the scaffold (highlighted in green) was virtually replaced using fragments derived by FDA-approved drugs. Panel B: drug-derived quinoline fragments were checked. The cinchocaine fragment Prestw-frag-3758 was highlighted in blue. This structure suggested the replacement of the ether linker in the quinoline family (e.g., inhibitor **1**) with an amide (represented in red). Panel C: virtual screening process and selected virtual hits. Virtual hits **4a**, **5a** and **6a** derived from the Prestwick fragments Prestw-frag-1560, Prestw-frag-2136 and Prest-frag-2303, respectively, that are highlighted in red. The chemical features of ModB and ModC are shown in green and yellow, respectively. ^a^ Fitness values.

Based on this background, compound **1** was selected as quinoline-representative leading structure for our scaffold hopping approach (Figure 2, panel A1), where new cores were selected from a library composed by 1456 small molecules (MW < 300) arising from the smart fragmentation of approved drugs [36].

The first core hopping run was aimed at replacing only the quinoline scaffold, while the two moieties on position 2 and 4 of the bicyclic system were completely preserved (run1: Figure 2, panels A1 and A2). In the second scaffold hopping experiment, the core definition included the quinoline nucleus together with the oxygen atom at position 4; this approach has been pursued with the aim of increasing the chemical diversity of the explored scaffolds (run2: Figure 2, panels A1 and A2).

The two independent scaffold hopping rounds were carried out using the ligand-based core hopping utility in Schrodinger [37]. The corresponding generated libraries were merged to obtain 6393 non redundant compounds defined as scaffold hopping set (Figure 2, panel C).

In parallel, a substructure search among the 1456 fragments using the quinoline core as query highlighted the presence of a fragment derived from the approved drug cinchocaine (the fragment is highlighted in blue in Figure 2, panel B). This compound shared a high chemical similarity with **1**, thus inspiring the modification of the ether linker in the quinoline compound with an amide to generate derivative **3a**. The latter compound was added to the previously described database to make up the scaffold hopping set, which was filtered in several rounds according to a funnel-like approach, each time discarding the molecules that did not meet the required criteria (Figure 2, panel C). 

In the first selection procedure, *in silico* absorption, distribution, metabolism and excretion (ADME) filters (see Material and methods) were applied to exclusively collect drug-like molecules. Second, the output compounds (1089 derivatives) were in turn virtually analyzed in Phase [38,39] by using the two pharmacophore models (i.e., ModB and ModC) as queries.

From each pharmacophore screening, only compounds showing a fitness score ≥1.7 were retained (723 and 292 compounds for ModB and ModC, respectively) and analyzed through a consensus approach by selecting only the compounds fitting both models. Additionally, compounds with a fitness higher than 2.0 for at least one of the two models were kept as well.

Third, the resulting compounds (167 molecules) were visually inspected looking for new compounds that had a novel scaffold and only the essential pharmacophore features. 

Taking into account the *in silico* results and an acceptable synthetic accessibility, four virtual hits (**3a**–**6a**, Figure 1 and Figure 2), each one having a different core, were considered worthy of experimental investigations as potential NorA EPIs. In particular, the virtual hit **6a** was designed starting from the scaffold hopping derivative **7** (Figure 2, panel C). The latter compound had a hydroxy-methyl group in position 4 of the pyridine ring. However, this substituent was not a pharmacophore element in the two models developed for NorA EPIs, and moreover its presence lowered the synthetic accessibility of the proposed compound. For this reason, the hydroxy-methyl group was removed retaining only the pyridine core to provide compound **6a**.

Furthermore, an additional set of 4 new compounds was designed by replacing the ethyl-*N*,*N*-diethylamine at position 4 in the previous series (a) with the 1-ethylpiperidine (**3b**–**6b**, Figure 1), that was the other well-characterized and promising chain in the quinoline chemical family (e.g., compound **2**).

## 3. Chemistry

**Scheme 1:**
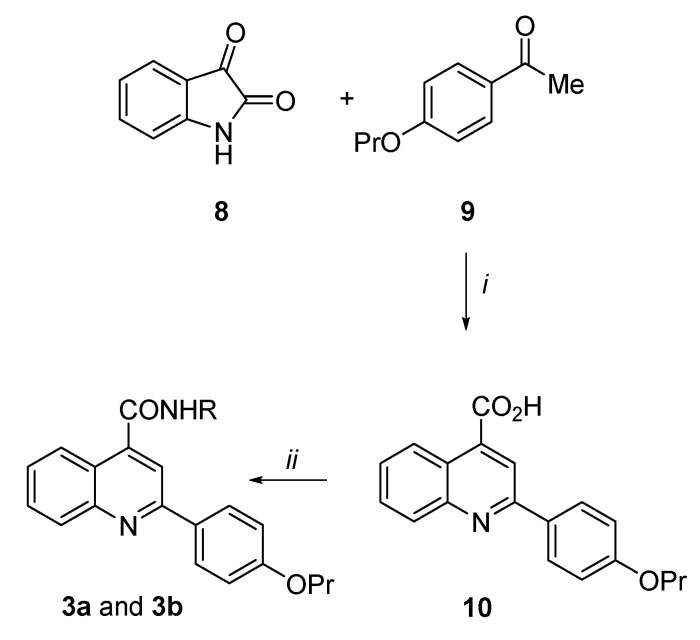
Reagents and condition: (***i***) KOH, EtOH, reflux, 7 h, 74%; (***ii***) *N*^1^,*N*^1^-diethylethane-1,2-diamine or 2-(piperidin-1-yl)ethan-1-amine, TBTU, DIPEA, dry DMSO, rt, 30 min–1 h, 26–46%.

**Scheme 2:**
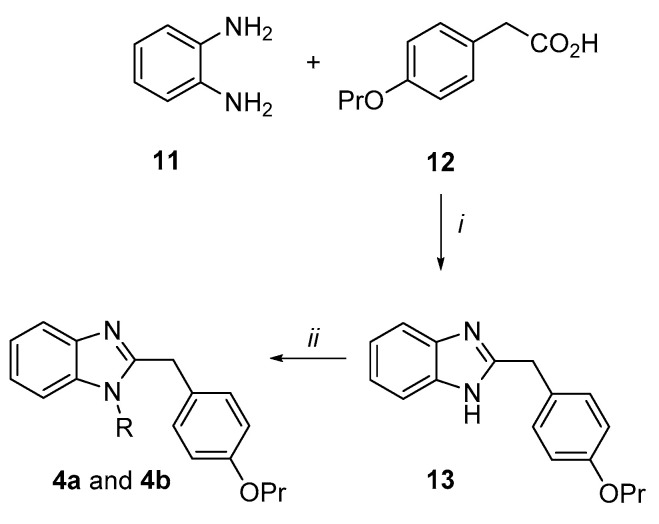
Reagents and condition: (***i***) H_3_BO_3_, dry toluene, reflux, 7 h, 91%; (***ii***) 2-chloro-*N*,*N*-diethylethan-1-amine hydrochloride or 1-(2-chloroethyl)piperidine hydrochloride, K_2_CO_3_, dry DMF, MW, 110 °C, 20–30 min, 29–35%.

**Scheme 3:**
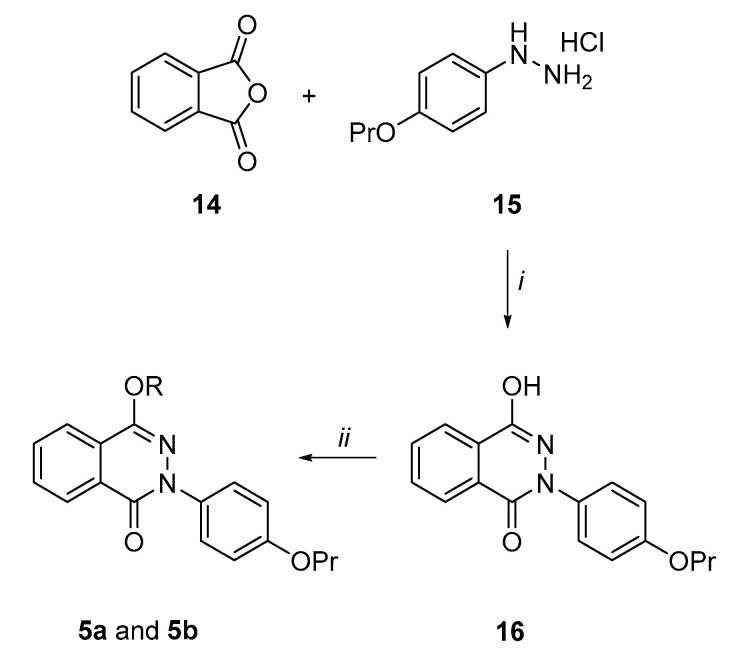
Reagents and condition: (***i***) CH_3_CO_2_H, reflux, 2 h, 13%; (***ii***) 2-chloro-*N*,*N*-diethylethan-1-amine hydrochloride or 1-(2-chloroethyl)piperidine hydrochloride, K_2_CO_3_, dry DMF, 90 °C, 2–3 h, 55%.

**Scheme 4:**
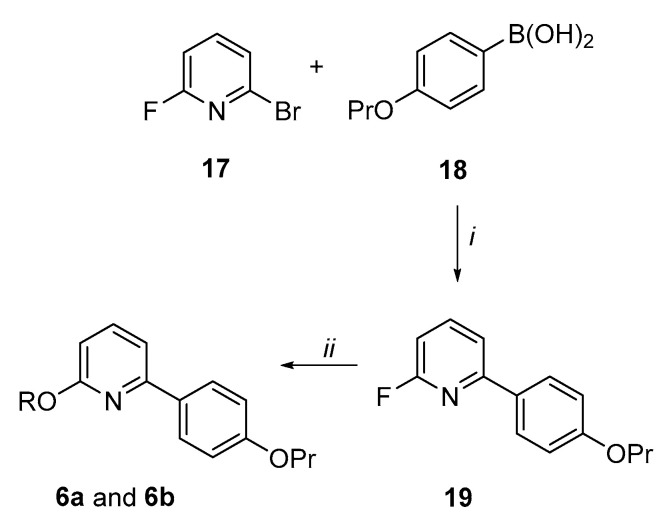
Reagents and condition: (***i***) Pd(PPh_3_)_4_, DME, 2M K_2_CO_3_, 80 °C, 5 h, 62%; (***ii***) 2-(diethylamino)ethan-1-ol or 1-piperidinethanol, NaH, dry THF, reflux, 1 h, 68–71%.

Synthetic procedures of the planned compounds **3a**, **3b**, **4a**, **4b**, **5a**, **5b**, **6a** and **6b** have been reported in Scheme 1, Scheme 2, Scheme 3 and Scheme 4.

**Synthesis of the 2-(4-propoxyphenyl)quinoline-4-carboxamide derivatives (3a and 3b) (Scheme 1).** By an analogue procedure reported by Liao et al. [40], isatin **8** was reacted with the 4-propoxy-acetophenone (**9**) in presence of KOH to give in good yields the intermediate 4-quinoline carboxylic acid (**10**). After coupling of **10** with the *N*^1^,*N*^1^-diethylethane-1,2-diamine or 2-(piperidin-1-yl)ethan-1-amine by using TBTU coupling reagent and DIPEA in dry DMSO at room temperature, the target carboxamide analogues **3a** and **3b** were obtained.

**Synthesis of the 2-(4-propoxybenzyl)-1*H*-benzimidazole derivatives (4a and 4b) (Scheme 2)**. *o*-Phenylenediamine **11** was condensed with the 4-propoxy-phenylacetic acid **12** in presence of a catalytic amount of boric acid in dry toluene by a Dean–Stark apparatus to give the benzimidazole derivative (**13**) in an excellent yield (91%). Subsequently, under microwave irradiation, **13** was reacted with 2-chloro-*N*,*N*-diethylethan-1-amine hydrochloride or 1-(2-chloroethyl)piperidine hydrochloride in dry DMF using K_2_CO_3_ as a base to afford the target benzimidazole analogues **4a** and **4b** in moderate yields.

**Synthesis of the 4-hydroxy-2-(4-propoxyphenyl)phthalazin-1(2*H*)-one derivatives (5a and 5b) (Scheme 3).** By following a similar procedure to Prime et al. [41], phthalic anhydride **14** was condensed in refluxing acetic acid with the hydrazine derivative (**15**), prepared according to the literature [42], to give the pthalazinone derivative (**16**), even though in low yields (13%). However, the reaction of **16** with 2-chloro-*N*,*N*-diethylethan-1-amine hydrochloride or 1-(2-chloroethyl)piperidine hydrochloride in dry DMF at 90 °C and using K_2_CO_3_ as a base afforded the target phthalazin-1(2*H*)-one derivatives **5a** and **5b** in quite good yields (55%).

**Synthesis of the 6-(4-propoxyphenyl)pyridin-2-ol derivatives (6a and 6b) (Scheme 4).** By Suzuki coupling conditions, 2-bromo-6-fluoropyridine (**17**) was reacted with 4-propoxy-phenylboronic acid (**18**) in a mixture of dimethoxyethane (DME) and aqueous 2M solution of K_2_CO_3_ in presence of a catalytic amount of Pd(PPh_3_)_4_ to give the 2-fluoro-6-(4-propoxyphenyl)pyridine **19** in a 62% yield. By aromatic nucleophilic substitutions, analogue **19** was reacted with 2-(diethylamino)ethan-1-ol or 1-piperidinethanol in presence of NaH in dry THF to afford the target pyridine derivatives **6a** and **6b** in good yields. 

## 4. Biological Results

For all synthesized derivatives (**3a**, **3b**, **4a**, **4b**, **5a**, **5b**, **6a** and **6b**), MIC assays (Table 1) were performed on two different *S. aureus* strains, SA-1199B (overexpressing *norA* gene and harboring a *grlA* mutation) [34] and SA-1199 (wild-type); compound **1** was included for comparative purpose.

**Figure 3:**
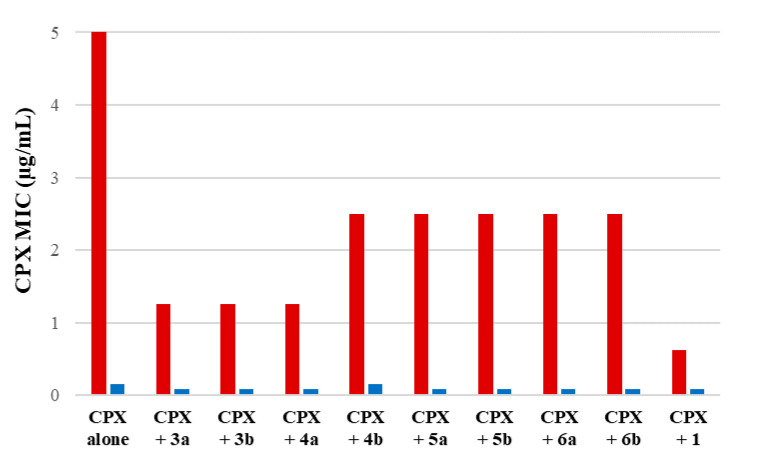
Synergistic assays of derivatives **3a**, **3b**, **4a**, **4b**, **5a**, **5b**, **6a** and **6b** and starting hit **1** at 12.5 µg/mL in combination with scalar concentrations of CPX against SA-1199B (red columns) and SA-1199 (blue columns).

None of the investigated compounds showed antibacterial activity against both *S. aureus* SA-1199 and SA-1199B at concentrations ≤25 µg/mL. Moreover, no significant difference can be observed between MIC values against SA-1199 and SA-1199B, the latter characterized by a higher amount of NorA protein than SA-1199. Thus, indirectly these data suggested that all compounds were not substrate of NorA efflux pump; indeed, a significant change in MIC values between SA-1199 and SA-1199B would be expected for a NorA substrate, as observed for CPX. Since all compounds showed MIC values ≥ 25 µg/mL, we proceeded to evaluate the synergistic effect of the derivatives at 12.5 µg/mL (a concentration ≤ ½ MIC for all compounds) in combination with scalar concentrations of CPX against both *S. aureus* strains (Figure 3). MIC values of CPX when tested alone against SA-1199 and SA-1199B can be found in Table 1 and Figure 3.

We considered promising EPIs those compounds that yielded a ≥4-fold decrease in the CPX MIC against SA-1199B and no significant change (≤2 times) against SA-1199. This difference in the synergistic activity with CPX between the two *S. aureus* strains is essential to display that the activity of compounds is related to NorA inhibition. Since *norA* gene is overexpressed in SA-1199B and normally expressed in SA-1199, compounds acting on NorA should possess a significant synergistic activity with CPX only against SA-1199B. Evident synergism also against SA-1199 could be likely due to a NorA-independent effect such as disruption, permeabilization or depolarization of the bacterial membrane, promoting the CPX penetration into bacterial cells.

**Figure 4:**
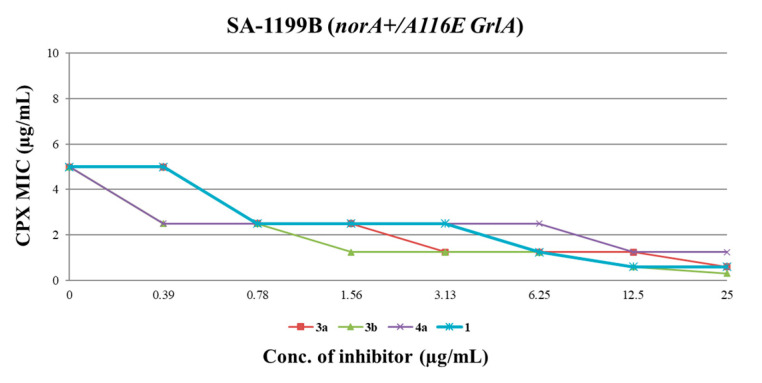
CPX MIC reduction against SA-1199B in the presence of increasing (doubling) concentrations of derivatives **3a**, **3b**, **4a** and starting hit **1**. A ≥ 4-fold MIC reduction was considered as synergism.

Focusing the attention on SAR, it was evident that the replacement of the quinolin-4-yloxy scaffold of **1** with the phthalazinone core (compounds **5a** and **5b**) led to a loss of the synergistic activity with the CPX against both *S. aureus* strains. Similarly, when the benzene moiety of the quinoline of **1** was removed to give the pyridine derivatives **6a** and **6b**, the synergistic activity was lost. On the other hand, quinoline-4-carboxamide derivatives (**3a** and **3b**) and only the benzimidazole **4a** displayed promising results by reducing, when tested at 12.5 µg/mL, the CPX MIC by 4-fold against SA-1199B while not producing a significant decrease in the CPX MIC against SA-1199. The lack of activity of the benzimidazole analogue **4b** was of more difficult interpretation. However, based on these results, we considered **3a**, **3b** and **4a** as interesting derivatives deserving further investigations. Thus, checkerboard assays were scheduled for all three derivatives in combination with CPX against SA-1199B (Figure 4).

All three compounds showed a dose-dependent synergistic effect in combination with CPX, exhibiting an activity similar or greater than that of the starting hit **1**. In particular, the worst of the three compounds (**4a**) retained a significant synergistic effect at concentrations ≥ 12.5 µg/mL, similarly to the starting hit **1**. On the other hand, carboxamide derivatives **3a** and **3b** exhibited better results, reducing the CPX MIC by 4-fold at concentrations as low as 3.13 and 1.56 µg/mL, respectively. These results are in agreement with the data obtained from EtBr efflux inhibition assays. Indeed, the carboxamide derivatives **3a** and **3b** showed a stronger ability to reduce the EtBr efflux on SA-1199B than the benzimidazole analogue **4a** (Table 2), with reductions up to 96–98% when used at 50 µM.

Overall, the obtained results show that the quinoline-4-carboxamide scaffold of **3a** and **3b** can efficiently replace the quinolin-4-yloxy nucleus, while the benzimidazoles resulted less interesting. In addition, data suggested that both derivatives **3a** and **3b** inhibited the NorA efflux pump without nonspecific effects. Indeed, synergism against the *norA*-overexpressing strain (SA-1199B) was observed for both compounds at concentration as low as 3.13 and 1.56 µg/mL (Figure 4) while no effect was detected against the wild type strain (SA-1199) using concentrations up to 12.5 µg/mL (Figure 3). The further demonstration that NorA could be inhibited by carboxamide analogues **3a** and **3b** was confirmed by their high degree of inhibition of the EtBr efflux observed through the EtBr efflux assays on SA-1199B. However, to rule out any doubt related to the mechanism of action of both derivatives, checkerboard assays were performed even against two specific *S. aureus* strains, SA-K1902 (*norA-*) and SA-K2378 (*norA++*). Since these two engineered strains are different only for the presence and expression level of the *norA* gene [43], it is expected that compounds that show a synergism with CPX only against SA-K2378 may inhibit the NorA efflux pump. On the other hand, those compounds having a significant synergism with CPX against both engineered strains should not be considered as NorA EPIs because they exhibit a synergistic activity not dependent on the presence of the NorA pump.

**Figure 5:**
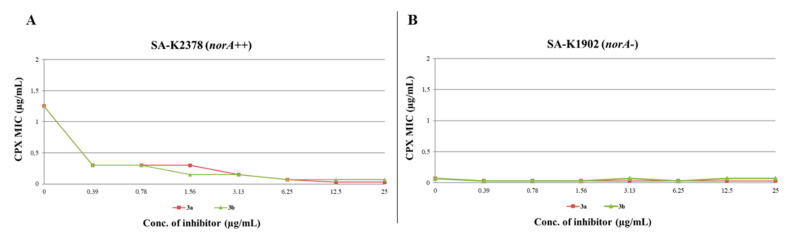
CPX MIC reduction in checkerboard assays of derivatives **3a** and **3b** in combination with CPX against (**A**) SA-K2378 (*norA++*) and (**B**) SA-K1902 (*norA-*). An antibiotic MIC reduction ≥ 4-fold indicated synergism.

Once excluded any antibacterial activity (MIC >25 µg/mL against SA-K2378 and SA-K1902), checkerboard assays against SA-K2378 (*norA++*) (Figure 5A) for compounds **3a** and **3b** highlighted, up to the lowest concentrations used (0.39 µg/mL), a significant synergism with CPX. On the other hand, as expected for NorA inhibitors, no significant synergistic effect with CPX was observed against SA-K1902 (*norA-*) (Figure 5B), thus confirming their “pure” activity against NorA. Remarkably, as a further demonstration that **3a** and **3b** were able to inhibit NorA, the CPX MIC against SA-K2378 in combination with EPIs never dropped below the MIC of the CPX tested alone against SA-K1902. This data is essential to prove that the synergistic activity of **3a** and **3b** serves to boost the CPX activity through the fully inhibition of the NorA efflux restoring the CPX MIC up to the levels observed in SA-K1902 in which *norA* is deleted.

Finally, we evaluated both compounds at 3.13 µg/mL, a concentration at which they reduced CPX MIC by 4-fold against SA-1199B, for their cytotoxic activity against human THP-1 and A549 (CCL-185^TM^) cell lines. Both exhibited a vitality higher than 50%, specifically THP-1 72% (**3a**) and 62% (**3b**) and A549 about 100% in presence of both compounds. In addition, it should be considered that SA-1199B is doubly resistant to CPX both for a single mutation on its target (DNA gyrase) and the overexpression of *norA* gene. Therefore, considering only the effect of the NorA inhibition observable on SA-K2378, both compounds at 0.39 µg/mL reduced the CPX MIC by 4-fold, thus showing that the inhibition of NorA occurs at concentrations significantly lower than those cytotoxic for human cells.

## 5. Material and Methods

### In Silico Scaffold Hopping

The Prestwick drug-fragment library was downloaded and submitted to LigPrep [44]. The neutral form of the ligands was prepared, and the tautomeric states was generated using Epik [45,46]. Furthermore, at most 32 stereoisomers per ligand and three lowest energy conformations per ligand ring were produced. Where not defined, all the chiral form of each stereocenter was produced. 

The ligand-based core hopping utility within Schrodinger was used to generate the scaffold-hopping libraries [37]. The input cores were generated starting from the prepared Prestwick drug-fragment library using the corefinder utility of Schrodinger [37], treating the entire fragment as core. 

The cores were filtered using the following criteria: number of heavy atoms ≤ 15; number of hydrogen bond acceptor ≤ 8; number of hydrogen bond donor ≤ 4; number of N+O ≤ 10; number of chiral centers = 0.

The library generated from the two scaffold hopping procedures were filtered using ADME properties calculated with QikProp [47]. The ligands were filtered applying the following criteria: QPlogP oct/wat, from −2 to 6.5; QPPCaco ≥500; QPlogKhsa, from −1.5 to 1.5; QPlogBB, from −3 to 1.2; Human oral absorption, ≥80%; Polar surface area (PSA), ≤200; #stars ≤1; rule of 5 ≤1; rule of three ≤1; #metabolite ≤5.

The remaining compounds were submitted to a conformational search using MacroModel [48]. To enhance the conformational sampling, the maximum number of steps was set to 10,000 per molecule. Conformers in an energy window of 5 kcal/mol were saved, discarding the redundant ones on the basis of their atomic rmsd (0.5 Å cutoff). Finally, the obtained conformers were screened in Phase [38,39] using ModB and ModC as queries.

## 6. Chemistry

All starting materials, reagents and solvents were purchased from common commercial suppliers and were used as such, unless otherwise indicated. Organic solutions were dried over anhydrous Na_2_SO_4_ and concentrated with a rotary evaporator at low pressure. The reactions carried out under MW irradiation were performed employing a microwave reactor BIOTAGE INITIATOR 2.0 version 2.3, build 6250. All reactions were routinely checked by thin-layer chromatography (TLC) on silica gel 60_F254_ (Merck) and visualized by using UV or iodine. Flash chromatography separations were carried out on Merck silica gel 60 (mesh 230–400) or by BUCHI Reveleris^®^ X2 Flash Chromatography (BÜCHI Labortechnik AG, Flawil, Switzerland). Melting points were determined in capillary tubes (Stuart SNP30, Stewart Italia, Milan, Italy) and are uncorrected. Yields were of purified products and were not optimized. ^1^H NMR spectra were recorded at 200 or 400 MHz (Bruker Avance DRX-200 or 400, respectively (Bruker Corporation, Massachusetts, USA)), while ^13^C NMR spectra were recorded at 101 MHz (Bruker Avance DRX-400). Chemical shifts are given in ppm (*δ*) relative to TMS. Spectra were acquired at 298 K. Data processing was performed with standard Bruker software XwinNMR (3.0) and the spectral data are consistent with the assigned structures. The purity of the tested compounds (≥95% sample purity) was evaluated by HPLC analysis using a Jasco LC-4000 instrument equipped with a UV-Visible Diode Array Jasco MD-4015 (Jasco Corporation, Tokyo, Japan) and an XTerra MS C18 Column, 5 µm, 4.6 mm × 150 mm (Waters Corporation, Massachusetts, USA). Chromatograms were analysed by ChromNAV 2.0 Chromatography Data System software.

*2-(4-propoxyphenyl)quinoline-4-carboxylic acid* (**10**). To a suspension of isatin **8** (0.69 g, 4.69 mmol) in KOH (33% *w*/*v*, 2 mL), a solution of 4-propoxyacetophenone **9** (1.00 g, 5.60 mmol) in EtOH (10 mL) was added and the mixture was refluxed for 7 h. After concentration of the solvent, the mixture was poured in ice/water, 2M HCl was added up to pH 2 and the precipitate obtained was filtered. After crystallization by Et_2_O/EtOH, the title compound **10** was obtained as a white solid in 74% yield, (m.p. 230.5–231.0 °C). ^1^H NMR (DMSO-*d*_6_, 200 MHz): δ 0.93 (3H, t, *J* = 7.3 Hz, OCH_2_CH_2_*CH*_3_), 1.61–1.79 (2H, m, OCH_2_*CH*_2_CH_3_), 3.95 (2H, t, *J* = 6.7 Hz, O*CH*_2_CH_2_CH_3_), 7.03 (2H, d, *J* = 8.8 Hz, H3′ and H5′), 7.59 (1H, dt, *J* = 1.7 and 8.3 Hz, H7), 7.76 (1H, dt, *J* = 1.8 and 7.5 Hz, H6), 8.05 (1H, d, *J* = 7.9 Hz, H5), 8.19 (2H, d, *J* = 8.4 Hz, H2′ and H6′), 8.35 (1H, s, H3), 8.55 (1H, d, *J* = 7.9 Hz, H8), 13.89 (1H, bs, CO_2_*H*).

*N-[2-(diethylamino)ethyl]-2-(4-propoxyphenyl)quinoline-4-carboxamide* (**3a**). Under N_2_ atmosphere, to a solution of derivative **10** (0.30 g, 0.98 mmol) in dry DMSO (3 mL), DIPEA (0.58 g, 4.19 mmol), TBTU (0.41 g, 1.27 mmol) and *N*^1^,*N*^1^-diethylethane-1,2-diamine (0.15 g, 1.27 mmol) were added and the reaction was stirred for 1 h at r.t. The mixture was poured in ice/water and extracted with EtOAc. The organic layers were washed with brine, dried over Na_2_SO_4_ and evaporated to dryness to give a brown oil. After purification by flash column chromatography eluting with CH_2_Cl_2_/MeOH 95/5, the title compound **3a** was obtained as a white solid in 45% yield, (m.p. 194.5–196.0 °C). ^1^H NMR (CDCl_3_, 400 MHz): δ 1.02–1.06 (9H, m, OCH_2_CH_2_*CH*_3_ and NCH_2_*CH*_3_ × 2), 1.79–1.87 (2H, m, OCH_2_*CH*_2_CH_3_), 2.58 (4H, q, *J* = 7.1 Hz, N*CH*_2_CH_3_ × 2), 2.74 (2H, t, *J* = 6.4 Hz, CONHCH_2_*CH*_2_N), 3.59–3.71 (2H, m, CONH*CH*_2_CH_2_N), 3.98 (2H, t, *J* = 6.6 Hz, O*CH*_2_CH_2_CH_3_), 6.99–7.03 (3H, m, NH, H3′ and H5′), 7.50 (1H, dt, *J* = 1.1 and 6.9 Hz, H6), 7.70 (1H, dt, *J* = 1.4 and 6.9 Hz, H7), 7.91 (1H, s, H3), 8.11–8.14 (3H, m, H8, H2′ and H6′), 8.21 (1H, d, *J* = 7.9 Hz, H5). ^13^C NMR (CDCl_3_, 101 MHz): δ 10.43, 11.52, 22.49, 37.20, 46.72, 51.29, 69.56, 114.78, 116.25, 123.08, 124.96, 126.65, 128.72, 129.86, 129.88, 131.25, 142.61, 148.77, 156.42, 160.59, 167.56. HPLC, CH_3_CN/0.1% diethylamine in H_2_O 50/50 to 90/10, retention time: 8.60 min.

*N-(2-piperidin-1-ylethyl)-2-(4-propoxyphenyl)quinoline-4-carboxamide* (**3b**). By following the procedure used to prepare compound **3a** and using 2-(piperidin-1-yl)ethan-1-amine (1.3 equiv.), the title compound **3b** was obtained as a white solid in 26% yield. Reaction time: 30 min; purification method: flash column chromatography eluting with CH_2_Cl_2_/MeOH 95/5, (m.p. 147.5–149.0 °C). ^1^H NMR (CDCl_3_, 400 MHz): δ 1.05 (3H, t, *J* = 7.4 Hz, OCH_2_CH_2_*CH*_3_), 1.38–1.45 (2H, m, piperidine CH_2_), 1.55–1.58 (4H, m, piperidine CH_2_ × 2), 1.78–1.88 (2H, m, OCH_2_*CH*_2_CH_3_), 2.40–2.51 (4H, m, piperidine NCH_2_ × 2), 2.61 (2H, t, *J* = 5.6 Hz, CONHCH_2_*CH*_2_N), 3.63–3.67 (2H, m, CONH*CH*_2_CH_2_N), 3.99 (2H, t, *J* = 6.6 Hz, O*CH*_2_CH_2_CH_3_), 6.77–6.85 (1H, bs, NH), 7.01–7.04 (2H, m, H3′ and H5′), 7.51 (1H, dt, *J* = 1.0 and 7.1 Hz, H6), 7.70 (1H, dt, *J* = 1.3 and 8.4 Hz, H7), 7.91 (1H, s, H3), 8.11–8.13 (3H, m, H8, H2′ and H6′), 8.21 (1H, d, *J* = 8.3 Hz, H5). ^13^C NMR (CDCl_3_, 101 MHz): δ 10.43, 22.49, 24.11, 25.71, 36.48, 54.23, 56.90, 69.57, 114.78, 116.38, 123.08, 124.91, 126.66, 128.74, 129.87, 129.89, 131.27, 142.68, 148.76, 156.44, 160.60, 167.55. HPLC, CH_3_CN/0.1% diethylamine in H_2_O 50/50 to 90/10, retention time: 8.88 min.

*2-(4-propoxybenzyl)-1H-benzimidazole* (**13**). To a solution of *o*-phenylenediamine **11** (0.21 g, 1.91 mmol) in dry toluene (20 mL), 4-propoxyphenylacetic acid **12** (0.56 g, 2.86 mmol) and H_3_BO_3_ (0.06 g, 0.19 mmol) were added and the resulting mixture was refluxed by a Dean–Stark apparatus for 7h. After concentration under vacuum of the solvent, a saturated solution of NaHCO_3_ was added and the resulting mixture was extracted with EtOAc. The organic layers were washed with brine, dried over Na_2_SO_4_ and evaporated to dryness to give a brown solid that was crystallized by Et_2_O/EtOH. After filtration, compound **13** was obtained as a light brown solid in 91% yield, (m.p. 187.0–188.0 °C). ^1^H NMR (DMSO-*d*_6_, 400 MHz): δ 0.91 (3H, t, *J* = 7.4 Hz, OCH_2_CH_2_*CH*_3_), 1.61–1.82 (2H, m, OCH_2_*CH*_2_CH_3_), 3.84 (2H, t, *J* = 6.5 Hz, O*CH*_2_CH_2_CH_3_), 4.06 (2H, s, benzylic CH_2_), 6.83 (2H, d, *J* = 8.5 Hz, H3′ and H5′), 7.07–7.09 (2H, m, Ar-H), 7.19 (2H, d, *J* = 8.8 Hz, H2′ and H6′), 7.42–7.44 (2H, m, Ar-H).

*N,N-diethyl-2-[2-(4-propoxybenzyl)-1*H*-benzimidazol-1-yl]ethanamine hydrochloride* (**4a**). Derivative **13** (0.50 g, 1.88 mmol), 2-chloro-*N*,*N*-diethylethan-1-amine hydrochloride (0.65 g, 3.76 mmol), K_2_CO_3_ (1.30 g, 9.40 mmol) and dry DMF (3 mL) were added in a MW vial. The mixture was irradiated by MW at the following conditions: time 20 min, max pressure 6 bar, cooling ON, temperature 110 °C. The mixture was then poured in ice/water, 10% NaOH was added up to pH 10 and EtOAc was used for the extraction. The organic layers were washed with brine, dried over Na_2_SO_4_ and evaporated to dryness to give a brown oil. After purification by flash column chromatography eluting with CH_2_Cl_2_/acetone 70/30, the title compound **4a** was obtained as a light oil that was dissolved in Et_2_O. After bubbling of HCl_gas_, compound **4a** was recovered as a white solid in 35% yield, (m.p. 118.0–119.5 °C). ^1^H NMR (DMSO-*d*_6_, 400 MHz): δ 0.92 (3H, t, *J* = 7.3 Hz, OCH_2_CH_2_*CH*_3_), 1.19 (6H, t, *J* = 7.2 Hz, NCH_2_*CH*_3_ × 2), 1.63–1.78 (2H, m, OCH_2_*CH*_2_CH_3_), 3.10–3.19 (4H, m, N*CH*_2_CH_3_ × 2), 3.25–3.31 (2H, m, NCH_2_*CH*_2_N), 3.87 (2H, t, *J* = 6.5 Hz, O*CH*_2_CH_2_CH_3_), 4.66 (2H, s, benzylic CH_2_), 5.02 (2H, m, N*CH*_2_CH_2_N), 6.92 (2H, d, *J* = 8.3 Hz, H3′ and H5′), 7.42 (2H, d, *J* = 8.1 Hz, H2′ and H6′), 7.47–7.57 (2H, m, H5 and H6), 7.74 (1H, d, *J* = 7.8 Hz, H4), 8.11 (1H, d, *J* = 7.9 Hz, H7), 12.13 (1H, bs, HCl). ^13^C NMR (DMSO-*d*_6_, 101 MHz): 8.69, 10.85, 22.44, 30.59, 39.34, 46.59, 48.48, 69.44, 113.30, 115.14, 115.47, 125.11, 126.04, 126.42, 131.08, 131.62, 132.25, 154.09, 158.77. HPLC, CH_3_CN/0.1% diethylamine in H_2_O 80/20, retention time: 3.58 min.

*1-(2-piperidin-1-ylethyl)-2-(4-propoxybenzyl)-1H-benzimidazole* (**4b**). By following the procedure used to prepare compound **4a** and using 1-(2-chloroethyl)piperidine hydrochloride (2.0 equiv.), the title compound **4b** was obtained as a white solid in 29% yield. Reaction time: 30 min; purification method: flash column chromatography eluting with CH_2_Cl_2_/acetone 90/10, (m.p. 104.0–105.0 °C). ^1^H NMR (DMSO-*d*_6_, 400 MHz): δ 0.99 (3H, t, *J* = 7.5 Hz, OCH_2_CH_2_*CH*_3_), 1.39–1.41 (2H, m, piperidine CH_2_), 1.53–1.56 (4H, m, piperidine CH_2_ × 2), 1.71–1.80 (2H, m, OCH_2_*CH*_2_CH_3_), 2.32–2.38 (6H, m, piperidine NCH_2_ × 2 and NCH_2_*CH*_2_N), 3.85 (2H, t, *J* = 6.6 Hz, O*CH*_2_CH_2_CH_3_), 4.08 (2H, t, *J* = 7.3 Hz, N*CH*_2_CH_2_N), 4.28 (2H, s, benzylic CH_2_), 6.80 (2H, d, *J* = 8.5 Hz, H3′ and H5′), 7.12 (2H, d, *J* = 8.5 Hz, H2′ and H6′), 7.19–7.29 (3H, m, H4, H5 and H6), 7.73–7.75 (1H, m, H7). ^13^C NMR (DMSO-*d*_6_, 101 MHz): δ 10.51, 22.54, 24.10, 25.90, 33.70, 41.83, 54.96, 57.51, 69.52, 109.26, 114.79, 119.52, 121.82, 122.18, 128.24, 129.47, 135.33, 142.68, 153.72, 158.10. HPLC, CH_3_CN/0.1% diethylamine in H_2_O 80/20, retention time: 3.73 min.

*4-Hydroxy-2-(4-propoxyphenyl)phtalazinon-1(2H)-one* (**16**). To a mixture of phthalic anhydride **14** (0.51 g, 3.45 mmol) in glacial acetic acid (5 mL), 4-propoxyphenylhydrazine hydrochloride **15** (0.79 g, 3.90 mmol) was added and the reaction mixture was refluxed for 2 h. After cooling, the reaction mixture was poured into ice/water and the brown precipitated was filtered. The crude solid was triturated by an aq. 1M Na_2_CO_3_ sol (100 mL) and the filtrate was acidified by 2N HCl to afford a white precipitate that, after filtration, gave the desired phtalazinone **16** as a low melting white solid in 13% yield. ^1^H NMR (400 MHz, CDCl_3_): δ 1.06 (3H, t, *J* = 7.5 Hz, OCH_2_CH_2_*CH*_3_), 1.90–1.75 (2H, m, OCH_2_*CH*_2_CH_3_), 3.98 (2H, t, *J* = 6.6 Hz, O*CH*_2_CH_2_CH_3_), 7.00–6.95 (2H, m, H3′ and H5′), 7.50–7.45 (2H, m, H2′ and H6′), 7.95–7.90 (2H, m, H6 and H7), 8.05–8.00 (1H, m, H5), 8.50 (1H, dd, *J* = 3.0 and 6.0 Hz, H8).

*4-[2-(Diethylamino)ethoxy]-2-(4-propoxyphenyl)phthalazin-1(2H)-one* (**5a**). Under N_2_ atmosphere, to a mixture of intermediate **16** (0.15 g, 0.51 mmol) and K_2_CO_3_ (0.28 g, 2.02 mmol) in dry DMF (4 mL), 2-chloro-*N,N*-diethylethanamine hydrochloride (0.17 g, 1.01 mmol) was added and the reaction mixture was heated at 90 °C for 3 h. After cooling, the reaction mixture was poured into ice/water, the aqueous mixture was extracted with EtOAc, the organic layers were washed with water, brine, dried over dry Na_2_SO_4_ and evaporated to dryness to give a crude oil. After purification by flash column chromatography eluting with CH_2_Cl_2_/MeOH 97/3, the title compound **5a** was obtained as a low melting white solid in 55% yield. ^1^H NMR (400 MHz, CDCl_3_): δ 1.00–1.25 (9H, m, OCH_2_CH_2_*CH*_3_ and NCH_2_*CH*_3_), 1.75–1.90 (2H, m, OCH_2_*CH*_2_CH_3_), 2.65 (4H, q, *J* = 7.1 Hz, N*CH*_2_CH_3_), 2.95 (2H, t, *J* = 6.0 Hz, OCH_2_*CH*_2_N), 3.95 (2H, t, *J* = 6.6 Hz, O*CH*_2_CH_2_CH_3_), 4.40 (2H, t, *J* = 6.0 Hz, O*CH*_2_CH_2_N), 6.92–7.00 (2H, m, H3′ and H5′), 7.55–7.60 (2H, m, H2′ and H6′), 7.75–7.85 (2H, m, H6 and H7), 7.98–8.02 (1H, m, H5), 8.40–8.50 (1H, m, H8); ^13^C NMR (101 MHz, CDCl_3_): δ 10.47, 11.97, 22.50, 47.86, 50.88, 65.50, 69.68, 114.23, 123.43, 124.64, 126.47, 127.52, 129.54, 131.90, 132.84, 134.87, 149.91, 157.86, 158.36. HPLC, CH_3_CN/0.1% diethylamine in H_2_O 70/30, retention time: 6.18 min.

*4-(2-Piperidin-1-ylethoxy)-2-(4-propoxyphenyl)phthalazin-1(2H)-one* (**5b**). By following the procedure used to prepare compound **5a** and using 1-(2-chloroethyl)piperidine hydrochloride (2.0 equiv.), the title compound **5b** was obtained as a yellow oil in 55% yield. Reaction time: 2h; purification method: flash column chromatography by eluting with CH_2_Cl_2_/MeOH 97/3. ^1^H NMR (400 MHz, CDCl_3_): δ 1.05 (3H, t, *J* = 7.4 Hz, OCH_2_CH_2_*CH*_3_), 1.40–1.50 (2H, m, piperidine *CH*_2_), 1.55–1.70 (4H, m, piperidine *CH*_2_ × 2), 1.75–1.90 (2H, m, OCH_2_*CH*_2_CH_3_), 2.50–2.65 (4H, m, piperidine N*CH*_2_ × 2), 2.85 (2H, t, *J* = 6.1 Hz, OCH_2_*CH*_2_N), 3.95 (2H, t, *J* = 6.6 Hz, O*CH*_2_CH_2_CH_3_), 4.45 (2H, t, *J* = 6.1 Hz, O*CH*_2_CH_2_N), 6.92–7.00 (2H, m, H3′ and H5′), 7.55–7.60 (2H, m, H2′ and H6′), 7.75–7.85 (2H, m, H6 and H7), 7.98–8.02 (1H, m, H5), 8.40–8.50 (1H, m, H8); ^13^CNMR (101 MHz, CDCl_3_): δ 10.44, 22.49, 24.06, 25.89, 54.89, 57.40, 64.90, 69.70, 113.96, 123.42, 124.64, 126.43, 127.51, 129.56, 131.86, 132.81, 134.89, 149.81, 157.86, 158.33. HPLC, CH_3_CN/0.1% diethylamine in H_2_O 70/30, retention time: 7.08 min.

*2-Fluoro-6-(4-propoxyphenyl)pyridine* (**19**). In three-necked round bottom flask, to a mixture of 2-bromo-6-fluoropyridine **17** (200 mg, 1.14 mmol), Pd(PPh_3_)_4_ (81 mg, 0.07 mmol), 4-propoxyphenylboronic acid **18** (252.73 mg, 1.4 mmol), in DME (3 mL), aq. 2M sol. of K_2_CO_3_ (1.14 mL, 2.28 mmol) was added. The reaction mixture was degassed under vacuum, filled with argon and heated at 80 °C. After 5 h, the reaction mixture was cooled to r.t, filtered over celite, and the filtrate was extracted with CHCl_3_. The organic layers were washed with brine, dried over dry Na_2_SO_4_ and evaporated to dryness to give a low melting solid that was purified by flash chromatography (Buchi X2 reveleris, eluting with cyclohexane/EtOAc, gradient 1% to 5% over 10 min) to afford the desired compound as low melting white solid in 62% yield. ^1^H NMR (400 MHz, CDCl_3_): δ 1.01 (3H, t, *J* = 7.5 Hz, OCH_2_CH_2_*CH*_3_), 1.75–1.90 (m, 2H, OCH_2_*CH*_2_CH_3_), 3.95 (2H, t, *J* = 6.6 Hz, O*CH*_2_CH_2_CH_3_), 6.77 (1H, dd, *J* = 3.0 and 8.1 Hz, H3), 6.93–6.97 (2H, m, H3′ and H5′), 7.53 (1H, dd, *J* = 2.5 and 7.6 Hz, H5), 7.77 (1H, q, *J* = 9.0 Hz, H4), 7.91–7.95 (2H, m, H2′ and H6′).

*N,N-diethyl-2-((6-(4-propoxyphenyl)pyridine-2-yl)oxy)ethan-1-amine* (**6a**). In a three-necked round bottom flask and under N_2_ atmosphere, to a mixture of 60% NaH (0.10 g, 2.64 mmol) in dry THF (5 mL), 2-(diethylamino)ethan-1-ol (0.30 g, 2.64 mmol) was added. After the end of bubbling, a solution of intermediate **19** (0.20 g, 0.86 mmol) in dry THF (5 mL) was added dropwise and the reaction mixture was stirred and at reflux. After 1 h, the reaction mixture was cooled at 0 °C with an ice bath, quenched with EtOAc and water, and then concentrated under vacuum. The aqueous mixture was extracted with EtOAc, the organic layers were washed with water, brine, dried over Na_2_SO_4_, and evaporated to dryness to give a yellow oil which was purified by flash chromatography (Buchi X2 reveleris, eluting with CH_2_Cl_2_/MeOH, gradient 0% to 10% over 30 min) to afford compound **6a** as a light yellow oil in 71% yield. ^1^H NMR (400 MHz, CDCl_3_): δ 0.95–1.15 (9H, m, OCH_2_CH_2_*CH*_3_ and NCH_2_*CH*_3_ × 2), 1.75–1.90 (2H, m, OCH_2_*CH*_2_CH_3_), 2.66 (4H, q, *J* = 7.1 Hz, N*CH*_2_CH_3_ × 2), 2.92 (1H, t, *J* = 6.4 Hz, OCH_2_*CH*_2_N), 3.95 (2H, t, *J* = 6.6 Hz, O*CH*_2_CH_2_CH_3_), 4.52 (1H, t, *J* = 6.4 Hz, OCH_2_*CH*_2_N), 6.60 (1H, d, *J* = 8.1 Hz, H3), 6.91–6.94 (2H, m, H3′ and H5′), 7.22 (1H, d, *J* = 7.5 Hz, H5), 7.54 (1H, t, *J* = 7.8 Hz, H4), 7.94–7.96 (2H, m, H2′ and H6′); ^13^C NMR (101 MHz, CDCl_3_): δ 10.49, 11.57, 22.52, 47.70, 51.25, 63.36, 69.47, 108.53, 111.84, 114.38, 127.85, 131.37, 139.08, 154.29, 159.84, 163.06. HPLC, CH_3_CN/0.1% diethylamine in H_2_O 70/30, retention time: 10.29 min.

*2-(2-(Piperidin-1-yl)ethoxy)-6-(4-propoxyphenyl)pyridine* (**6b**). By following the procedure used to prepare compound **6a** and using 1-piperidinethanol (3.0 equiv.) as nucleophile, the title compound **6b** was obtained as a yellow oil in 68% yield. Reaction time: 1 h; purification method: flash chromatography (Buchi X2 reveleris, eluting with CH_2_Cl_2_/MeOH, gradient 0 to 10% over 15 min). ^1^H NMR (400 MHz, CDCl_3_): δ 1.00 (3H, t, *J* = 7.5 Hz, OCH_2_CH_2_*CH*_3_), 1.40–1.50 (2H, m, piperidine CH_2_), 1.52–1.68 (4H, m, piperidine CH_2_ × 2), 1.75–1.90 (2H, m, OCH_2_*CH*_2_CH_3_), 2.43–2.60 (4H, m, piperidine N*CH*_2_CH_3_ × 2), 2.80 (1H, t, *J* = 6.4 Hz, OCH_2_*CH*_2_N), 3.95 (2H, t, *J* = 6.6 Hz, O*CH*_2_CH_2_CH_3_), 4.55 (2H, t, *J* = 6.1 Hz, OCH_2_*CH*_2_N), 6.60 (1H, d, *J* = 8.1 Hz, H3), 6.98–6.90 (2H, m, H3′ and H5′), 7.22 (1H, d, *J* = 7.5 Hz, H5), 7.56 (1H, t, *J* = 7.8 Hz, H4), 7.97–7.93 (2H, m, H2′ and H6′); ^13^C NMR (101 MHz, CDCl_3_): δ 10.46, 22.52, 24.21, 25.88, 54.98, 57.90, 63.10, 69.50, 108.52, 111.77, 114.41, 127.84, 131.44, 139.00, 154.31, 159.84, 163.15. HPLC, CH_3_CN/0.1% diethylamine in H_2_O 70/30, retention time: 10.17 min.

## 7. Bacterial Strains, Cell Lines, Culture Media and Antibiotics

The *S. aureus* strains used in this work included SA-1199 (wt) [34], SA-1199B (overexpressing *norA* and also possessing an A116E GrlA substitution) [34], SA-K1902 (*norA*-deleted) [43] and SA-K2378. The last strain was derived from SA-K1902 transformed with a recombinant plasmid obtained by cloning the SA-1199B *norA* sequence and its promoter into the multicopy plasmid pCU1. All *S. aureus* strains were cultured in Triptone Soy Agar or Mannitol Salt agar plates and maintained in Triptone Soy Broth supplemented with 20% glycerol at −80 °C. Chloramphenicol (10 µg/mL) was added to the above-mentioned media when growing the strains SA-K1902 and SA-K2378. All culture media were purchased from Oxoid (Oxoid S.p.A., Rodano, Milano, Italy).

The human monocytes cell line THP-1 was purchased from ATCC (American Type Culture Collection), while the A549 (CCL-185^TM^) cell line was kindly provided by Dr. Tatiana Armeni (Polytechnic University of Marche, Ancona, Italy). Cells were maintained respectively in Roswell Park Memorial Institute (RPMI-1640) or in a 50:50 mixture of Dulbecco′s Modified Eagle′s Medium (DMEM) and Ham’s F12 medium (F12), both from Corning Incorporated (Corning, New York, NY, USA), supplemented with 10% fetal bovine serum (FBS, Corning Incorporated), 1% L-glutamine and 2% antimitotic/antibiotic, at 37 °C in a humid atmosphere containing 5% CO_2_.

All antibiotics were from Sigma-Aldrich (Saint Louis, MO, USA). 

## 8. EtBr Efflux

*S. aureus* cells (SA-1199B) were grown overnight in cation-supplemented Mueller−Hinton broth (SMHB) containing no additive at 35 °C. Organisms were diluted into the same medium as used for exponential growth until an optical density at 600 nm (OD_600_) of 0.7−0.8 was achieved. Cells were then pelleted and resuspended at OD_600_ = 0.8 in 0.5 mL aliquots of SMHB containing EtBr plus carbonyl cyanide m-chlorophenylhydrazone (CCCP) to “load” cells with EtBr (final concentrations, 25 μM for EtBr and 100 μM for CCCP). After 20 min at rt, cells were pelleted then resuspended in 1 mL of fresh SMHB, and 200 μL aliquots were immediately transferred into the wells of opaque 96-well plates containing no, 10 or 50 μM test compound. Fluorescence was monitored continuously using a BioTek FLx800 microplate reader (BioTek Instruments Inc., Winooski, VT, USA) at excitation and emission wavelengths of 485 and 645 nm, respectively, for 5 min. Experiments were conducted in triplicate performing two technical replicates for each biological replicate. Efflux activity of SA-1199B was expressed as fluorescence decrease (%) over a 5 min time course. Efflux inhibition was determined using the equation ([efflux in the absence] − [efflux in the presence of test [compound])/[efflux in the absence of test compound] × 100, giving the percent efflux inhibition observed.

## 9. Antimicrobial Susceptibility Assays

The antimicrobial activity of the compounds and of their combinations with CPX was evaluated by broth microdilution MIC determination against the isogenic pair SA-1199 and SA-1199B using doubling concentrations of the tested drug, according to the CLSI guidelines [49]. Checkerboard assays were performed as previously described [28], considering synergistic the combinations leading to a ≥4-fold reduction of the CPX MIC [50]. All assays were performed in duplicate; when the results did not overlap, the higher MIC value was reported.

## 10. Cytotoxicity Assays

The cytotoxic effect of the compounds was determined by MTT assays performed on THP-1 and A549 (CCL-185^TM^) cells after 24 h exposure [51]. Cells were seeded at the density of 1 × 10^4^ cells/well into 96-well flat-bottomed plates in 200 µL of RPMI or DMEM/F12 medium, respectively, and then exposed for 24 h to the compounds at the concentrations resulted synergistic in checkerboard assays. Unexposed cells were used as negative control. The colorimetric MTT assay allowed to measure the cell growth rates through the amount of the accumulated intracellular insoluble formazan crystals, subsequently dissolved using DMSO and quantified spectrophotometrically (OD_570_), using a microplate reader (BioTeK, Winooski, VT, USA). The obtained data were analyzed by the software Gen05 v3. The percentage of viable cells was calculated as follows: % Cell viability = 100 × Experimental well absorbance/untreated control well absorbance. All assays were performed in biological and technical duplicate.

## 11. Conclusions

In this work, *in silico* scaffold-hopping approaches combined with 3D-pharmacophore screening followed by chemical synthesis and validated biological methods allowed for the identification of two quinoline-4-carboxamide derivatives (**3a** and **3b**) as new NorA EPIs. Compounds **3a** and **3b** were able to synergize at low concentrations with CPX against the *norA* overexpressing *S. aureus* strains SA-1199B and SA-K2378. We also proved that the synergistic effect of our EPIs was due to a NorA inhibition, indirectly ruling out potential nonspecific effects by EtBr efflux inhibition on SA-1199B and checkerboard assays on the isogenic pairs SA-K2378 (*norA++*) and SA-K1902 (*norA-*). Moreover, through the synthesis and biological evaluation of different compounds, we observed some important SAR information revealing that the quinoline core is essential to retain NorA inhibition; however, functionalization at the C-4 position of this nucleus with a carboxamide moiety (instead of an ethereal function as for the starting hit **1**) led to an improvement of both the synergistic activity with CPX and the cytotoxicity profile against human cell lines. Based on these promising results, virtual hits that have been discarded from this work, because they are less synthetically accessible but promising in terms of fitness scores, will be considered for future experimental validations.

## Figures and Tables

**Table 1:** MIC values (µg/mL) of derivatives **3a**, **3b**, **4a**, **4b**, **5a**, **5b**, **6a**, **6b**, the starting hit **1** and CPX against SA-1199 and SA-1199B.

	*S. aureus* Strains	
**Compd.**	SA-1199	SA-1199B
**3a**	25	25
**3b**	>50	50
**4a**	>50	>50
**4b**	>50	>50
**5a**	>50	>50
**5b**	>50	>50
**6a**	50	>50
**6b**	50	25
**1**	50	25
**CPX**	0.16	5

**Table 2:** EtBr efflux inhibition (%) on SA-1199B exerted by compounds **3a**, **3b** and **4a**.

Compd.	EtBr Efflux Inhibition (%)	
	50 µM	10 µM
**3a**	98.1	56.6
**3b**	96.4	66.2
**4a**	74.5	35.8
